# The MR Imaging of Primary Intrahepatic Lymphoepithelioma-like Cholangiocarcinoma: A Diagnostic Challenge

**DOI:** 10.3390/diagnostics13182998

**Published:** 2023-09-20

**Authors:** Yangyang Liu, Dajing Guo, Xiaojing He, Xi Liu, Weijie Chen, Lingli Chen, Yuan Ji, Mengsu Zeng, Mingliang Wang

**Affiliations:** 1 Department of Radiology, The Second Affiliated Hospital of Chongqing Medical University, Chongqing 400010, China; liuyy@cqmu.edu.cn (Y.L.); guodaj@hospital.cqmu.edu.cn (D.G.); he_xiaojing@hospital.cqmu.edu.cn (X.H.); liuxi@camu.edu.cn (X.L.); 2 Department of Cardiology, The Second Affiliated Hospital of Chongqing Medical University, Chongqing 400010, China; cqmucwj@hospital.cqmu.edu.cn; 3 Department of Pathology, Zhongshan Hospital, Fudan University, Shanghai Geriatric Medical Center, Shanghai 200032, China; chen.lingli@zs-hospital.sh.cn (L.C.); ji.yuan@zs-hospital.sh.cn (Y.J.); 4 Department of Radiology, Zhongshan Hospital, Fudan University, Shanghai Geriatric Medical Center, Shanghai 200032, China; zengmengsu@outlook.com

**Keywords:** intrahepatic lymphoepithelioma-like cholangiocarcinoma, magnetic resonance imaging, imaging features, EBV infection

## Abstract

Purpose: To characterize the magnetic resonance imaging features of primary intrahepatic lymphoepithelioma-like cholangiocarcinoma (LELCC). Materials and Methods: Thirty-four patients with 38 histologically confirmed LELCCs were enrolled retrospectively from January 2014 to August 2022. We evaluated the clinical features, histologic findings, and imaging manifestations on dynamic enhanced MRI. Results: 74% (25/34) of the cases were associated with EBV infection. Moreover, patients infected with EBV exhibited a lower level of Ki-67 proliferation. The serum CA199 level was elevated in 10 patients. The median tumor diameter was 2.8 cm (range, 1.1–8.7 cm). Most tumors were well-defined with a smooth or lobulated margin and showed peripheral hyperintensity and central hypointensity on T2-weighted imaging (T2WI). T2 hyperintense foci were recognized in 8 patients. In the dynamic enhanced MRI, 21 tumors demonstrated Type A enhancement pattern (rim enhancement), 10 demonstrated Type B (rapid wash-in and wash-out), and seven demonstrated Type C (rapid wash-in without wash-out). Capsular enhancement in PVP or DP was found in 22 tumors. A few patients had satellite lesions, portal vein thrombosis, bile duct dilatation, and distal metastasis. Lymph node metastases were discovered pathologically in 11 patients. Conclusions: MRI findings of LELCC vary and are non-specific. While a majority of LELCCs exhibit typical features of intrahepatic cholangiocarcinoma (iCCA), unique findings like T2 hyperintense foci or capsular enhancement could suggest LELCC. EBV infection and elevated tumor markers can aid in differentiation. However, given the mimics of some cases of liver hypervascular lesions, histological examination remains essential for definitive diagnosis.

## 1. Introduction

Lymphoepithelioma-like carcinoma (LELC) is a rare malignant epithelial tumor characterized histologically as undifferentiated carcinoma with a significant amount of lymphoplasmacytic infiltration [1,2,3]. This tumor can appear in various parts of the body, including the liver, stomach, esophagus, lung, thymus, skin, breast, etc. The majority of cases are reported to be associated with the Epstein–Barr virus (EBV) infection, which is prevalent in Asia.

Primary LELC of the liver can be classified as lymphoepithelioma-like hepatocellular carcinoma (LEL-HCC) [4] and lymphoepithelioma-like cholangiocarcinoma (LELCC) [5]. LELCC is a relatively rare and peculiar subtype of intrahepatic cholangiocarcinoma (iCCA), which accounts for less than 5% of all iCCAs [6]. Nevertheless, its clinical features, imaging manifestations, and prognosis differ from those of typical iCCAs [7,8].

As of 28 April 2023, over 150 cases have been reported, but only a few of these described the magnetic resonance imaging (MRI) features [9,10,11,12,13]. Among them, the study conducted by Pan et al. was the largest, including 37 LELC patients with 34 LELCCs [14]. However, their study primarily utilized the Liver Imaging Reporting and Data System (LI-RADS) category to predict the post-surgery prognosis of LELC. It offered a limited assessment of MRI features specific to LELCCs. The next largest study on MRI findings for LELLC comprised only six cases [15]. This research emphasized the EBV-positive cases of LELCCs and did not include EBV-negative ones, suggesting a potential variance in results. The remaining reported cases were mainly described from a clinical perspective, emphasizing histologic and immunohistochemical findings with limited discussion on imaging features [6,9,10,12,13,16].

Thus, we conducted a retrospective study examining the MRI manifestations of all cases in our institution. To date, this is the most comprehensive study focusing on the MRI features of LELLC, enhancing the understanding of the disease.

## 2. Material and Methods

### 2.1. Patients

This retrospective study was approved by our institutional board, and informed consent was waived, while written informed consent was obtained from all patients before they took the MR exam. We retrospectively searched the institution’s pathology database between January 2014 and August 2022 with histologically diagnosed LELCC using surgical resection. Forty-two patients with LELCCs were histologically diagnosed after surgical resection. Inclusion criteria: (1) dynamic enhanced liver MRI examination within one month before surgery; (2) no treatment for liver lesion before MRI examination. Exclusion criteria: (1) no classification between LELCCs and LEL-HCCs; (2) history of a primary tumor in the liver; (3) history of nasopharyngeal carcinoma or the possibility of a metastasis from primary nasopharyngeal carcinoma.

### 2.2. Imaging Acquisition

All imaging was performed on either a 1.5 T (Siemens MAGNETOM Avanto, Erlangen, Germany and United Imaging Healthcare uMR 560, Shanghai, China) or a 3.0 T MRI scanner (United Imaging Healthcare uMR 770, Shanghai, China and Siemens Verio, Erlangen, Germany). The routine MRI protocol included the following sequences: an axial T2-weighted imaging (T2WI) with fat saturation (fs), an axial in-phase and out-phase T1-weighted imaging (T1WI), an unenhanced axial fs T1WI and dynamic triple-phase contrast-enhanced MRI. Diffusion-weighted imaging (DWI) was performed using a breath-hold single-shot echo-planar imaging pulse sequence with b values of 0 and 500 mm^2^/s.

Contrast-enhanced MRI was performed with gadopentetate dimeglumine (Magnevist®, Bayer Schering Pharma, Berlin, Germany) (*n* = 32) or gadoxetic acid (Primovist ®, Bayer Schering Pharma, Berlin, Germany) (*n* = 2) via a power injector (Spectris Solaris^®^ EP MR, MEDRAD Inc., Indianola, IA, USA) at an infusion rate of 1.5–2 mL/s. After injection of the contrast agent, a three-dimensional fs T1W gradient-echo sequence was used to acquire dynamically enhanced images in the arterial phase (AP), portal venous phase (PVP), and delayed phase (DP) at 25–30 s, 60–80 s, and 150–180 s, respectively. In addition, for the two patients who received gadoxetic acid-enhanced MRI, hepatobiliary phase images were obtained 10–15 min after the contrast administration.

### 2.3. Imaging Analysis

The MR images were reviewed on the institutional PACS workstation (Centricity Radiology RA1000, GE Healthcare, Barrington, IL, USA). Two experienced radiologists, one with more than 15 years of and one with 10 years of subspecialty experience in abdominal MRI, independently evaluated the MR images. A consensus decision was reached if there was divergence.

The following imaging features were acquired from the MR images, including tumor location, whether beneath the liver capsule or not, largest cross-sectional tumor diameter, tumor shape (round or oval vs. irregular) and contour (well defined or obscure), appearance of the liver capsule (bulging, retraction, partial bulging, and retraction, neither bulging nor retraction), lesion texture (hypointense, intermediate hypointense, isointense on T1WI, homogenously intermediate/hyperintense, peripheral hyperintensity and central hypointensity, heterogeneous, isointense on T2WI, presence of T2WI hyperintense foci, whether the lesion is lobulated or not, dynamic enhancement pattern, capsular enhancement, dilatation of intrahepatic biliary ducts, presence of satellite nodules, and presence of lymph node metastasis or distant metastases. The MRI reports were also reviewed to determine the original radiologic diagnosis of each case at the moment of the procedures. Tumors were classified by the AJCC (the American Joint Committee on Cancer) TNM staging systems. Lesions in patients with HBV or chronic hepatic diseases were also categorized according to the LI-RADS v2018.

In the dynamic enhanced MRI, tumors were classified based on their enhancement patterns into three categories:

(i) Type A: Rim enhancement, characteristic of typical iCCA.
(ii) Type B: Nonrim arterial phase hyperenhancement with wash-out in PVP or DP, which resembles the “rapid wash-in and wash-out” seen in typical HCC.
(iii) Type C: Nonrim arterial phase hyperenhancement with either consistent enhancement or isointensity in PVP or DP, or “rapid wash-in without wash-out”

The lesions that did not show any of these three patterns were concluded into the unclassified enhancement pattern.

### 2.4. Statistical Analysis

All data were analyzed statistically using SPSS version 23.0 (IBM, Armonk, NY, USA). Quantitative data were expressed as the mean ± SD (standard deviation) and categorical variables as numbers (percentages). Differences between groups for continuous variables were assessed using the Student’s *t*-test (Gaussian distribution), while categorical variables were assessed using the chi-square test or Fisher’s exact test. Variables with *p* < 0.05 in univariate analyses were included in the multivariate analysis.

## 3. Results

### 3.1. Clinical Findings

From January 2014 to August 2022, 42 LELCCs were pathologically diagnosed after surgical resection. Thirty-four patients (16 males and 18 females) with 38 lesions were included in this study; 8 patients were excluded because of a lack of MR imaging. Most of the tumors were incidentally detected during routine health examinations, with only three patients presenting with upper abdominal pain and one with back pain. The clinical characteristics are shown in Table 1. The median age of the total 34 patients was 58 years (range 38 to 82 years). 74% (25/34) have EBV infection, which was diagnosed based on positive EBER in situ hybridization results. Additionally, the result showed that EBV infection is correlated with the Ki-67 index. Twenty patients had chronic hepatitis of cirrhosis. HBV status was positive in 15 patients (44%), and cirrhosis was recognized in 9 patients. No patients had a history of chronic HCV.

The serum CA199 level was elevated in 10 patients (31%). The median CA199 of the 10 elevated patients was 107.9 ku/L, ranging from 31.6 ku/L to 695.5 ku/L. Among them, one patient has a slight elevation of alpha-fetoprotein (AFP, 25.4μg/L). AFP variant (26%) and Protein Induced using Vitamin K Absence or Antagonist-II (PIVKA-II, 9552 mAU/mL) levels were elevated in two patients because one or more synchronous HCC was found in the liver. In one patient with synchronous multiple tumors (two HCCs and three LELCCs), both the elevation of the AFP variant (21%) and PIVKA-II (60 mAU/mL) were recognized without the elevation of CA199. None of the patients have elevated CEA.

### 3.2. Imaging Manifestations

The imaging manifestations of LELCC in individual patients are shown in Table 2. Twenty-nine patients (29/34) had only a solitary lesion, and five patients had more than one lesion. Moreover, four out of five patients with multiple lesions had one or more coexisting HCCs, whereas only one patient had all his lesions be LELCCs. Twenty-two tumors were located in the right lobe, 15 tumors in the left lobe, one tumor in both left and right lobes, and none in the caudate lobe. The median tumor diameter was 2.8 cm (range, 1.1–8.7 cm). Twenty-two tumors were beneath the capsule. Sixteen were round, 11 were lobulated, and 11 were irregular. Seventeen tumors were lobulated. Most of the tumors (25/38) were well-defined, and 13 were with an obscure margin. Seven tumors had a bulging appearance of the liver capsule, two showed retraction, and five had both bulging and retraction of the liver capsule.

**Figure 1:**
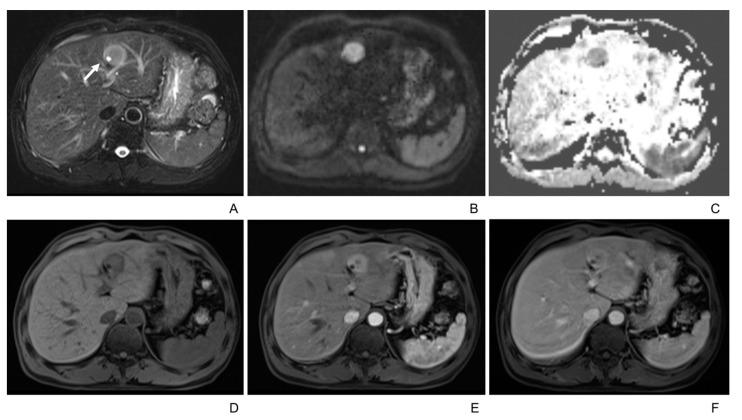
49-year-old male with intrahepatic lymphoepithelioma-like cholangiocarcinoma. (**A**) T2-weighted MR image displays a well-defined hyperintense tumor in the left lobe of the liver, with hyperintense foci inside it (white arrow). (**B**,**C**) Tumor shows hyperintensity on diffusion-weighted imaging (b = 500 m^2^/s) and hypointensity on the ADC map. (**D**–**F**) Tumor shows hypointensity in plain scan and rapid wash-in without wash-out (Type C enhancement pattern) in the arterial and portal venous phase.

On T1WI, six lesions were significantly hypointense, 30 were intermediate hypointense, and two were isointense or not visible. On T2WI, eight were homogenously intermediate or hyperintense,18 showed peripheral hyperintensity and central hypointensity, and 12 were heterogeneous. T2 hyperintense foci were recognized in eight patients, and half of them had more than one foci (Figure 1). On DWI, 17 lesions were significantly hyperintense (2 were hyperintense in the periphery region), and 21 were intermediate hyperintense (4 were hyperintense in the periphery region). The mean ADC value was 1070 ± 255 mm^2^/s, and that of the liver was 1335 ± 351 mm^2^/s. The difference was statistically significant (*p* < 0.05). Necrosis was recognized in 5 lesions. None of the tumors were found to have fat or lipids inside.

**Figure 2:**
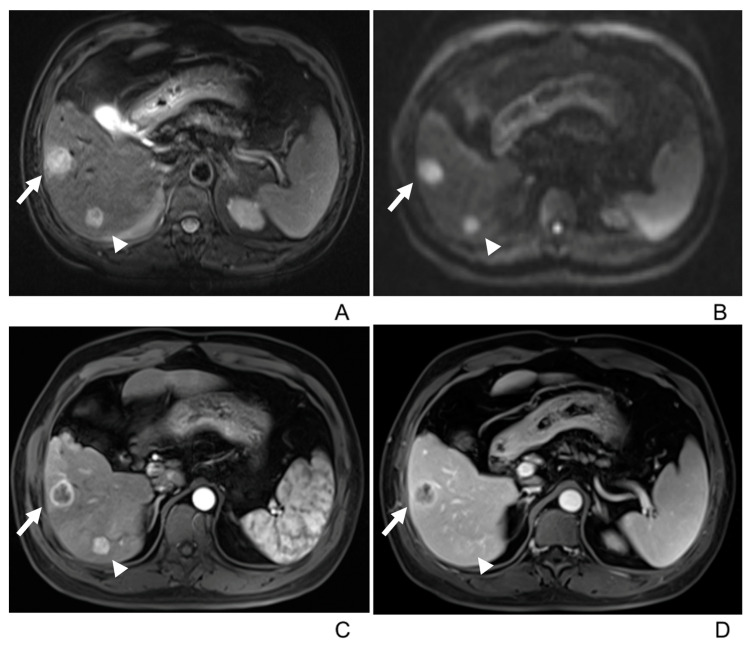
51-year-old male with multiple intrahepatic lymphoepithelioma-like cholangiocarcinomas (>2). (**A**) T2-weighted MR image displays two well-defined hyperintense tumors in the right lobe. (**B**) Both tumors show hyperintensity on diffusion-weighted imaging (b = 500 m^2^/s). (**C**,**D**) On the arterial and portal venous phase images, the tumor in segment V (arrow) shows peripheral rim arterial-phase enhancement with centripetal progressive enhancement (Type A enhancement pattern), and the tumor in segment VI (arrowhead) shows rapid wash-in and rapid wash-out (Type B enhancement pattern), respectively.

**Figure 3:**
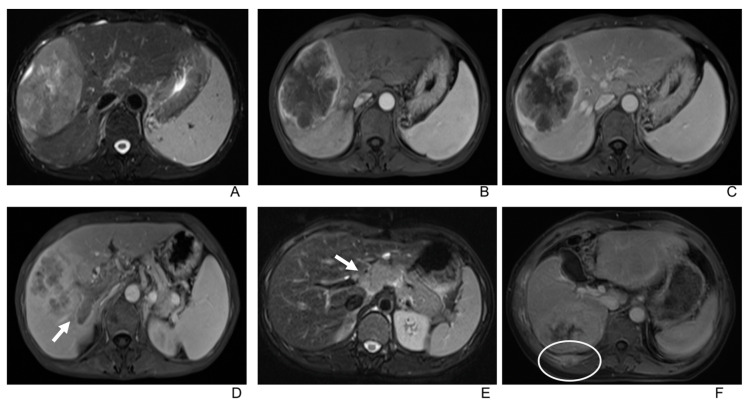
(**A**) T2-weighted imaging shows uneven hyperintensity of a large intrahepatic lymphoepithelioma-like cholangiocarcinoma in a 55-year-old male. (**B**,**C**) Tumor shows peripheral rim enhancement in the arterial phase with centripetal progressive enhancement (Type A enhancement pattern) in the portal venous phase. (**D**) Tumor thrombosis in the right branch of the portal vein (arrow). (**E**) Enlarged lymph node in the portal hepatis (arrow) in a 39-year-old female. (**F**) Metastasis in the 12th rib (circle) of a 56-year-old male.

**Figure 4:**
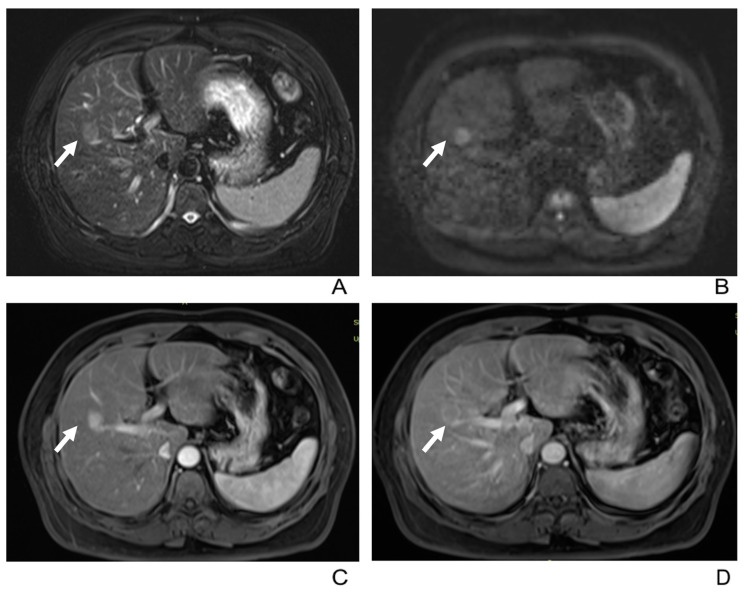
52-year-old male with intrahepatic lymphoepithelioma-like cholangiocarcinoma. (**A**) T2-weighted MR image displays a well-defined hyperintense tumor (arrow) in the right lobe of the liver. (**B**) Tumor (arrow) shows hyperintensity on diffusion-weighted imaging (b = 500 m^2^/s). (**C**,**D**) Tumor (arrow) shows “rapid wash-in and rapid wash-out (Type B enhancement pattern) in the arterial and portal venous phase and delayed capsular enhancement in the portal venous phase.

In the dynamic enhanced MRI, 21 tumors demonstrated Type A enhancement pattern (Figure 2 and Figure 3), 10 demonstrated Type B (Figure 2 and Figure 4), and 7 demonstrated Type C (Figure 1) (Table 3). There was no significant correlation between gender, age, or tumor size and the enhancement patterns in both univariate and multivariate analyses.

Capsular enhancement in PVP or DP was found in 22 tumors that were confirmed to be pseudo-capsules based on the histology, except for one tumor, which had an incomplete capsule (Figure 4). This capsular enhancement was observed in a significant number of tumors with a Type A pattern (13/21) and almost all tumors with a Type B pattern (9/10). However, Type C tumors did not exhibit this feature.

Additional observations include:

- Only two lesions showed a scar-like enhancement in the tumor in AP.
- Two tumors had associated satellite lesions.
- Four patients had portal vein tumor thrombosis (Figure 3).
- Three patients exhibited intrahepatic biliary dilation, and one patient had distal metastasis on the 12th rib (Figure 3).
- Furthermore, lymph node enlargement was found in 9 patients, and metastases were discovered pathologically in 11 patients. The median short-axial diameter of the lymph node was 2.1 cm (range, 0.7 cm to 2.9 cm). Only two lymph nodes had necrosis inside, while the others all showed homogeneous hypervascular enhancement.
- Two patients underwent the gadoxetic acid-enhanced MRI, and both tumors showed hypointense in the hepatobiliary phase.

According to the AJCC TNM staging, 28 patients were classified as stage I; three patients were stage II, one was stage III, and two were stage IV. Eleven patients were N1. One patient is M1. The lesions were classified into three categories in patients with HBV infection or chronic liver diseases, according to LI-RADS v2018. Four lesions were classified into LR-4 (probably HCC), 8 were LR-5 (definitely HCC), and 8 were LR-M (probably or definitely malignant but not HCC specific) (Table 3). All the patients underwent surgery, with two receiving TACE and three receiving PD-1 downstaging therapy before surgery, respectively.

## 4. Discussion

In the retrospective study, we discerned three distinct enhancement patterns in the lesions, categorized as Type A (rim enhancement), Type B (rapid wash-in and wash-out), and Type C (rapid wash-in without wash-out). Over half of the LELCCs displayed the Type A enhancement pattern, typically seen in iCCAs. Yet, we also noted atypical findings like T2 hyperintensity foci, which are typically associated with HCC, angiomyolipoma, or capsular enhancement. These observations may lean the diagnosis more towards LELCC than iCCA, especially in patients with EBV infections. Notably, we observed no gender differences in the study population, encompassing both EBV-positive and EBV-negative patients.

In assessing the three enhancement patterns, our observations diverge from previous studies predominantly centered on EBV-positive cases. Only a few cases of EBV-negative cases were reported, with limited information on imaging features [17,18]. These earlier investigations of EBV-positive cases predominantly focused on CT findings and identified a Type B enhancement pattern in most lesions [9,10,15,16]. Reports on MRI features are inadequate. Liver lesions might exhibit diverse enhancement patterns on MRI compared to CT features. Only Ding et al. [10]. and Liao et al. [9]. reported two cases that both showed the Type A enhancement pattern on MRI. Hur et al. [11] described a centrifugal enhancement pattern in PVP, whereas Aosasa et al. [19] delineated only the unenhanced scan findings of a solitary case on MRI. Remarkably, within our EBV-positive cohort, the Type A enhancement pattern was still the most predominant, followed by Type B and Type C in a ratio of 14:8:5. This distribution complicates the differential diagnosis with typical iCCA, particularly given the distinct prognostic implications—LELCC generally exhibits a more favorable prognosis than iCCA [7]. The lesions characterized by the Type B enhancement pattern closely resemble the typical HCC, marked by rapid wash-in and wash-out, thus presenting diagnostic challenges [20,21]. Those with a type C enhancement pattern further pose differentiation difficulties, especially when compared to other hypervascular liver lesions such as focal nodular hyperplasia (FNH), hepatic adenoma (HCA), and lymphoma. Notably, in certain instances, some large lesions exhibit an absence of necrosis.

In patients with HBV or chronic liver diseases, the categorization of LELCCs using LI-RADS v2018 displays variability. Over half of the LELCCs were classified as non-LR-M. Of these, 20% (4/20) were classified as LR-4, 40% (8/20) as LR-5, and the remaining 40% (8/20) as LR-M. Therefore, when evaluating a lesion with a background of chronic liver disease, LI-RADS may not be optimal for differentiating LELCC from its mimics. However, it can offer crucial prognostic insights. Pan et al. [14] previously assessed the classification of LELC (including LEL-HCC and LELCC) into either LR m or LR-4/5, aiming to identify factors influencing recurrence-free survival. Their findings highlighted a significant correlation between the LI-RADS category and the postsurgical prognosis of LELC, with tumors labeled as LR m exhibiting poorer recurrence-free survival than those classified as LR-4/5 [14].

In our study, over half of the cases exhibited capsular enhancement. Among these, Type A was the predominant pattern, followed by Type B, while type C was not observed. Capsular enhancement is more commonly seen in HCC than in iCCA [22]. The presence of capsular enhancement in the Type A enhancement pattern may suggest the diagnosis of LELCC. However, when it appears in Type B, differentiation from HCC becomes more challenging. While prior studies have reported central scar-like enhancement [23], it was infrequent in our findings, identified in just two cases.

Interestingly, T2 hyperintense foci were found in under a third of the tumors (10/38), with more frequent occurrence in males (6/10). Although these findings were mentioned in prior literature, they remain uncommon [15]. Such cystic foci are seldom seen in iCCA but have been reported in HCC [22] and hepatic epithelioid angiomyolipoma [24]. The emergence of these cystic foci might result from endothelial damage of the sinusoid due to increased intratumor pressure within encapsulation or hemorrhage inside the lesion [22,25,26]. Therefore, when a lesion presents classic iCCA features with internal cystic foci, the diagnosis of LELCC may be suggested.

Other features like satellite lesions, portal vein thrombosis, bile duct dilatation, and distal metastasis were observed but were not common. Tumors were hypointense in the hepatobiliary phase, with no contrast retention observed. Lymph node enlargement was seen in fewer than one-third of the patients. All metastatic lymph nodes exhibited hyperenhancement, with only a minority showing necrosis. Importantly, even small lymph nodes showing homogeneous hyperenhancement could also be metastatic lymph nodes.

No gender difference was found in our study, despite previous literature reporting a female predominance [10,15]. The ratio of men to women was approximately equal, with slightly more women than men. This difference is likely due to a selection bias, as most reported LELCC patients were EBV-positive. When we focused only on those with EBV-positive cases, the ratio shifted more towards women, with a ratio of 18:7.

Our study observed a similar incidence of EVB infection in primary LELCC patients as reported in previous studies [5,9,10,15,16,27,28,29,30,31]. Approximately 74% of the patients presented with EBV infection, with one case showing a marginal rise in peripheral blood EBV level. We identified a significant correlation between EBV infection and the Ki-67 proliferation. Specifically, patients with EBV infection exhibited a lower Ki-67 proliferation rate than those without the infection. Given that Ki-67 is a trusted marker for cell proliferation [28], elevated expression levels of Ki-67 are often linked to poorer prognoses and increasing tumor grades [29,32]. This observation offers insight into the potentially improved prognosis seen in LELCC compared to iCCA, warranting further research.

More than half of the cases (58.8%) had chronic hepatitis or cirrhosis, with 15 cases being HBV positive and nine cases exhibiting cirrhosis. This aligns with previous studies reporting a high prevalence of HBV carriers in China, a country with a significant HBV infection burden [15]. According to available literature, 45.9% of Chinese LELCC patients associated with EBV were HBV-positive [5,9,10,16,27,28,29,30,31]. We assume that this is related to HBV’s endemic status in China. The relationship between HBV and EBV infection and LELCC development remains uncertain.

The majority of patients presented with a single lesion, with one-third showing elevated CA199 levels. This differs from the previous study [15], which suggested that tumor marker elevations were uncommon. Elevated HCC-related tumor markers were rare. In patients with multiple lesions, most (4/5) were found to have synchronous HCCs and LELCCs. All of these patients exhibited increased levels of HCC-related tumor markers, such as AFP, AFP-L3, or PIVKA-III. Thus, elevated HCC-related tumor markers in patients with multiple lesions might suggest the coexistence of HCC.

LELCC is predominately located in the right lobe of the liver. Around half of the tumors exhibit peripheral hyperintensity and central hypointensity on T2WI, followed by a significant number showing heterogeneous intensity and a minority presenting homogenously intermediate/hyperintense. Notably, our data did not find a correlation between tumor size and T2 signal patterns, which deviates from earlier research [15]. While larger tumors often show heterogeneity, a subset can manifest as homogenous intermediate hyperintensity on T2WI, complicating differentiation from lymphoma. Almost all the tumors appear hypointensity on T1WI. DWI and ADC maps showed diffusion restriction within these tumors, aiding in lesion detection and potentially indicating malignancy. Yet, the specific utility of DWI and ADC maps in differentiating LELCC from other similar lesions requires further investigation.

This study has several limitations. It is retrospective and recruited patients based on surgical results, possibly introducing sampling bias. Additionally, it only focused on imaging features of LELCCs without comparing them to other liver hypervascular diseases. Therefore, further cohort studies are needed to explore differences in diagnosis and prognosis among different diseases.

## 5. Conclusions

In conclusion, MRI findings of LELCCs vary and lack specific imaging signs. More than half of the cases demonstrate typical iCCA-like imaging features and enhancement patterns. Additional findings, such as T2 hyperintense foci or capsular enhancement, can assist in differential diagnosis. EBV infection and elevated tumor markers may also help in the differentiation diagnosis. However, the preoperative identification of LELCCs remains intricate, owing to their resemblance to HCC, FNH, or HCA. Hence, histological assessment remains indispensable for a definitive diagnosis.

## Figures and Tables

**Table 1:** Patient Demographics.

Parameter	
Mean age (range)	58 y (38 y, 82 y)
Sex (M:F)	16:18
EBER (+)	25/34 (74%)
Chronic hepatitis or cirrhosis	20/34 (58.8%)
Hepatitis B (+)	15/34 (44%)
Cirrhosis	9/34 (26.5%)
CA199 (+)	10/32 * (31%), median 52.08 ku/L, range (45 ku/L, 695.5 ku/L)
AFP(>20 μg/L)	1/34 (3%,), range (25.4 μg/L)
AFP variant	2/34 (6%), range (21%, 26%)
PIVKA-II	1/34 (3%), range (9552 mAU/mL)

* 2 without record.

**Table 2:** MRI Characteristics of LELLC.

Parameter	*n* (%)
Location	
Left lobe	15 (40%)
Right lobe	22 (58%)
Left and right lobe	1 (3%)
Caudate lobe	0
In peripheral subcapsular region	22 (58%)
Tumor shape (round, oval, or irregular)	(16/11/11)
Lesion contour	
Well defined	25 (66%)
Obscure	13 (34%)
Appearance of liver capsule	
Bulging	7 (18%)
Retraction	2 (5%)
Partial bulging and retraction	5 (13%)
Neither bulging nor retraction	21 (55%)
T1WI	
Hypointense	6 (16%)
Intermediate hypointense	30 (79%)
Isointense/not seen	2 (5%)
T2WI	
Homogenously intermediate/hyperintense	8 (21%)
Peripheral hyperintensity and central hypointensity	18 (47%)
Heterogeneous	12 (32%)
Isointense/not seen	0
T2 hyperintense foci	10 (26%)
Lobulated	17 (45%)
Cirrhosis on imaging	3 (8%)
ADC value of lesions (mm^2^/s)	1070 ± 255
ADC value of liver (mm^2^/s)	1335 ± 351
	*p* = 0.001 (<0.05)
AJCC staging (34)	
T1	28
T2	3
T3	1
T4	2
N1	11
M1	1
LI-RADS v2018 (20)	
LR-4	4
LR-5	8
LR-M	8

Note—T1WI: T1-weighted imaging, T2WI or T2: T2 weighted imaging, ADC: apparent diffusion coefficient, AJCC: American Joint Committee on Cancer, T: tumor, N: lymph node, M: metastasis, LI-RADS: Liver Imaging Reporting and Data System, LR: LI-RADS.

**Table 3:** Enhancement Characteristics of LELLC.

Parameter	LELLC
Dynamic enhancement patterns	
Type A ^a^	21(55%)
Type B ^b^	10(26%)
Type C ^c^	7(18%)
Capsular enhancement	22/38(58%)
Type A/Type B/Type C	13/9/0/(62%/90%/0)
Satellite lesion	2/38(6%)
Venous tumor thrombus	4/38(11%)
Biliary dilatation	3/38(8%)
Lymphadenopathy	9/34(26%)
Distal metastasis on ribs	1/34(3%)

^a^: Rim enhancement, characteristic of typical iCCA. ^b^: Nonrim arterial phase hyperenhancement with wash-out in PVP or DP, which resembles the “rapid wash-in and wash-out” seen in typical HCC. ^c^: Nonrim arterial phase hyperenhancement with either consistent enhancement or iso-intensity in PVP or DP, or “rapid wash-in without wash-out”.

## Data Availability

The datasets are available only on request due to privacy/ethical restrictions and can be requested from the corresponding author.
